# A bottom-up model of functional outcome in schizophrenia

**DOI:** 10.1038/s41598-021-87172-4

**Published:** 2021-04-07

**Authors:** Hongge Luo, Yanli Zhao, Fengmei Fan, Hongzhen Fan, Yunhui Wang, Wei Qu, Zhiren Wang, Yunlong Tan, Xiujun Zhang, Shuping Tan

**Affiliations:** 1 School of Public Health, North China University of Science and Technology, Tangshan, China; 2 College of Psychology, North China University of Science and Technology, Tangshan, China; 3 Beijing Huilongguan Hospital, Peking University Huilongguan Clinical Medical School, Beijing, China

**Keywords:** Psychology, Risk factors

## Abstract

Schizophrenia results in poor functional outcomes owing to numerous factors. This study provides the first test of a bottom-up causal model of functional outcome in schizophrenia, using neurocognition, vocal emotional cognition, alexithymia, and negative symptoms as predictors of functional outcome. We investigated a cross-sectional sample of 135 individuals with schizophrenia and 78 controls. Using a series of structural equation modelling analyses, a single pathway was generated among scores from the MATRICS Consensus Cognitive Battery (MCCB), vocal emotion recognition test, Toronto Alexithymia Scale (TAS), Brief Negative Symptom Scale, and the Personal and Social Performance Scale. The scores for each dimension of the MCCB in the schizophrenia group were significantly lower than that in the control group. The recognition accuracy for different emotions (anger, disgust, fear, sadness, surprise, and satire, but not calm was significantly lower in the schizophrenia group than in the control group. Moreover, the scores on the three dimensions of TAS were significantly higher in the schizophrenia group than in the control group. On path analysis modelling, the proposed bottom-up causal model showed a strong fit with the data and formed a single pathway, from neurocognition to vocal emotional cognition, to alexithymia, to negative symptoms, and to poor functional outcomes. The study results strongly support the proposed bottom-up causal model of functional outcome in schizophrenia. The model could be used to better understand the causal factors related to the functional outcome, as well as for the development of intervention strategies to improve functional outcomes in schizophrenia.

## Introduction

Schizophrenia is characterized by positive and negative symptoms and widespread deficits in neurocognition^1^, social cognition^2^, and functional outcome^3^. Functional outcome is one of the most important evaluation indexes of recovery from schizophrenia. In fact, measurements of functional outcome are highly correlated with the degree of neurocognitive impairment in schizophrenia^4^. However, increasing evidence suggests that social cognition mediates the relationship between neurocognition and functional outcome^5–8^, including working performance, vocational outcomes^9^, independent living and social functioning^10^, as well as quality of life^11^. Additionally, a previous meta-analysis found that functional outcome was more strongly associated with social cognition than with neurocognition^12^. Social cognition is a broad field encompassing the psychological operations of perceiving as well as interpreting and processing information to adapt to social interactions. The most widely studied aspects of social cognition in schizophrenic patients include emotional processing, social perception, attrition bias, and mentalizing^13^.

Emotional processing plays a critical role in schizophrenia research, as patients often display aberrant emotional response, expression, and recognition. Emotional recognition is crucial in interpersonal communication as it refers to the ability of individuals to effectively recognize other people’s emotions, through facial expressions as well as the prosodic components of vocal communication. Emotional processing is believed to involve at least two separate processes, namely, vocal expressions processed through cortical-based mechanisms associated with perception and effortful stimulus evaluation, and facial expressions processed through subcortical emotional and mnemonic mechanism^14^. Previous studies have suggested that facial expressions represent an effective way to convey basic or simple emotions, while vocal expressions may be more suitable for communicating complex emotional information^15^, such as attitude and social intention^16^ and satire^17^. Therefore, the ability to recognize vocal emotions is an important component of social competence^15^ and an organic combination of emotion and language. Previous research has largely focused on the recognition of facial emotion; thus, there are few studies examining the role of vocal emotion recognition in schizophrenia. However, given the important role that vocal emotion recognition plays in social cognition, we aimed to further clarify its relationship with neurocognition and functional outcome in schizophrenia.

Another example of impaired social cognition that commonly occurs in patients with schizophrenia is alexithymia, an emotional disorder characterized by the inability to identify and describe one’s emotions^18,19^. Individuals with a high degree of alexithymia have difficulty recognizing facial expressions, especially those with negative valence^20^. However, the relationship between alexithymia and vocal emotional recognition remains unclear. Some studies reported a relationship^21,22^, yet others have failed to find one^23^. Moreover, the presence of alexithymia has been identified to predict poorer social and daily functioning, exceeding the contribution of previously determined factors, including neurocognition and social cognition (e.g. theory of mind and emotion recognition)^24^. Therefore, exploring the relationship between them is warranted.

The presence of negative symptoms, which refers to a lack of and/or decrease in normal behaviour and subjective experience, including blunted affect, alogia, anhedonia, avolition, and asociality, has long been considered a hallmark of schizophrenia^25^. Importantly, these symptoms were related to social cognition, including the recognition of facial emotion and theory of mind^26^, and were recently found to be related to alexithymia^27^. However, the connection between alexithymia and negative symptoms has received little empirical support. Furthermore, the presence of negative symptoms is a strong predictor of a range of poor clinical outcomes^28^, including more serious functional impairments^29^, poorer psychosocial functioning^30,31^, poorer life skills and quality of life^32^, lower subjective well-being^33^, lower recovery rates^34^, and poor prognosis and response to medication^35^.

As a result, impairments in social cognition and the presence of negative symptoms are both core features of schizophrenia and are closely associated with poorer functional outcomes. However, the specific factors and pathways that lead to functional impairment are still unclear. In general, previous research has focused mainly on the neurocognitive deficits and negative symptoms, and often failed to simultaneously examine other relevant factors^36^. Therefore, whether these important outcome factors lie on one or multiple pathways is unknown. For the first time, we used structural equation modelling to simultaneously evaluate a bottom-up causal model characterizing the pathways among them in a population of Chinese schizophrenia patients. We hypothesized that these variables would form a single pathway, from neurocognition to vocal emotional cognition to alexithymia to negative symptoms to poor functional outcomes.

## Results

### Demographic information, cognition, symptoms, and functional results

Table 1 shows demographic information and other scale scores of the participants included in the study. There were no significant group differences in age or education, but there was a significant difference in gender (*x*^2^ = 15.52, *p* = 0.000) between patients and controls. A total of 61% of the patients scored between 31 and 70 on the Personal and Social Performance Scale (PSP), suggesting that a majority of the patients had moderate to severe functional impairment. The scores for each dimension of the MATRICS Consensus Cognitive Battery (MCCB) were significantly lower in the schizophrenia group than in the control group. Additionally, the recognition accuracy of different emotions (anger, disgust, fear, sadness, surprise, and satire, but not for calm (*t* = -1.28, *p* = 0.201)) in the schizophrenia group was significantly lower than that in the control group. Compared with other emotions, the accuracy of disgust was the lowest in the patients (*F* = 111.78, *p* = 0.000). Finally, the scores for the two dimensions and total scores of the TAS were also significantly higher in the schizophrenia group than in the control group (detailed in Table 1).

**Table 1:** Comparisons of demographic, neurocognition, vocal emotion cognition, alexithymia, negative symptoms and functioning variables between two groups.

	Schizophrenia group(n = 135)	Health group(n = 73)	*Statistics* *t or x*^2^	*P*
Gender	Male = 83 Female = 52	Male = 24 Female = 49	15.52	0.000
Age(years, mean ± SD)	43.45 ± 11.72	40.35 ± 9.96	1.91	0.057
Education(years, mean ± SD)	13.06 ± 2.38	13.64 ± 2.14	-1.75	0.082
Duration of Illness(year)	19.44 ± 11.23			
**PSP**	67.77 ± 10.80			
Fraction range (n/%)	0–30 (0/0%)			
	31–70 (82/61%)			
	71–100(53/39%)			
**BNSS Total**	23.36 ± 12.83			
Anhedonia	5.71 ± 3.81			
Distress	1.47 ± 1.35			
Asociality	4.30 ± 2.35			
Avolition	3.99 ± 2.60			
Blunted affect	4.50 ± 3.72			
Alogia	2.63 ± 2.44			
**MCCB Total**	45.95 ± 9.84	56.14 ± 6.80	-7.89	0.000
Verbal learning	46.97 ± 10.67	55.82 ± 8.31	-6.15	0.000
Reasoning and problem solving	46.21 ± 11.68	56.06 ± 8.39	-6.37	0.000
Visual learning	45.65 ± 10.36	51.99 ± 8.39	-4.42	0.000
Social cognition	46.74 ± 9.83	49.53 ± 10.20	-0.550	0.583
Attention	46.14 ± 10.38	55.71 ± 7.54	-6.95	0.000
Processing speed	46.22 ± 9.57	57.41 ± 7.38	-8.69	0.000
working memory	48.36 ± 10.17	56.80 ± 7.38	-6.25	0.000
**Voice emotion cognition Total**	29.31 ± 10.03	37.38 ± 7.21	-6.07	0.000
anger	5.58 ± 1.99	6.64 ± 1.45	-4.04	0.000
calm	7.01 ± 1.67	7.28 ± 1.11	-1.28	0.201
disgust	2.53 ± 2.11	3.89 ± 1.90	-4.58	0.000
fear	3.80 ± 2.63	4.73 ± 2.02	-2.62	0.001
sadness	4.27 ± 2.09	6.19 ± 1.60	-6.86	0.000
satire	3.03 ± 2.31	3.97 ± 2.24	-2.84	0.005
surprise	3.10 ± 2.25	4.67 ± 2.15	-4.90	0.000
**TAS Total**	50.30 ± 10.08	44.45 ± 9.50	4.07	0.000
F1	16.42 ± 5.31	13.01 ± 5.06	4.49	0.000
F2	12.85 ± 3.35	10.97 ± 2.86	4.06	0.000
F3	21.03 ± 3.33	20.47 ± 3.07	1.20	0.233

PSP: Personal and Social Performance Scale.

BNSS: Brief Negative Symptom Scale.

MCCB: MATRICS Consensus Cognitive Battery.

TAS: Toronto Alexithymia Scale.

F1: Difficulty identifying feelings.

F2: Difficulty describing feelings.

F3: Externally oriented thinking.

### Measurement model

**Figure 1:**
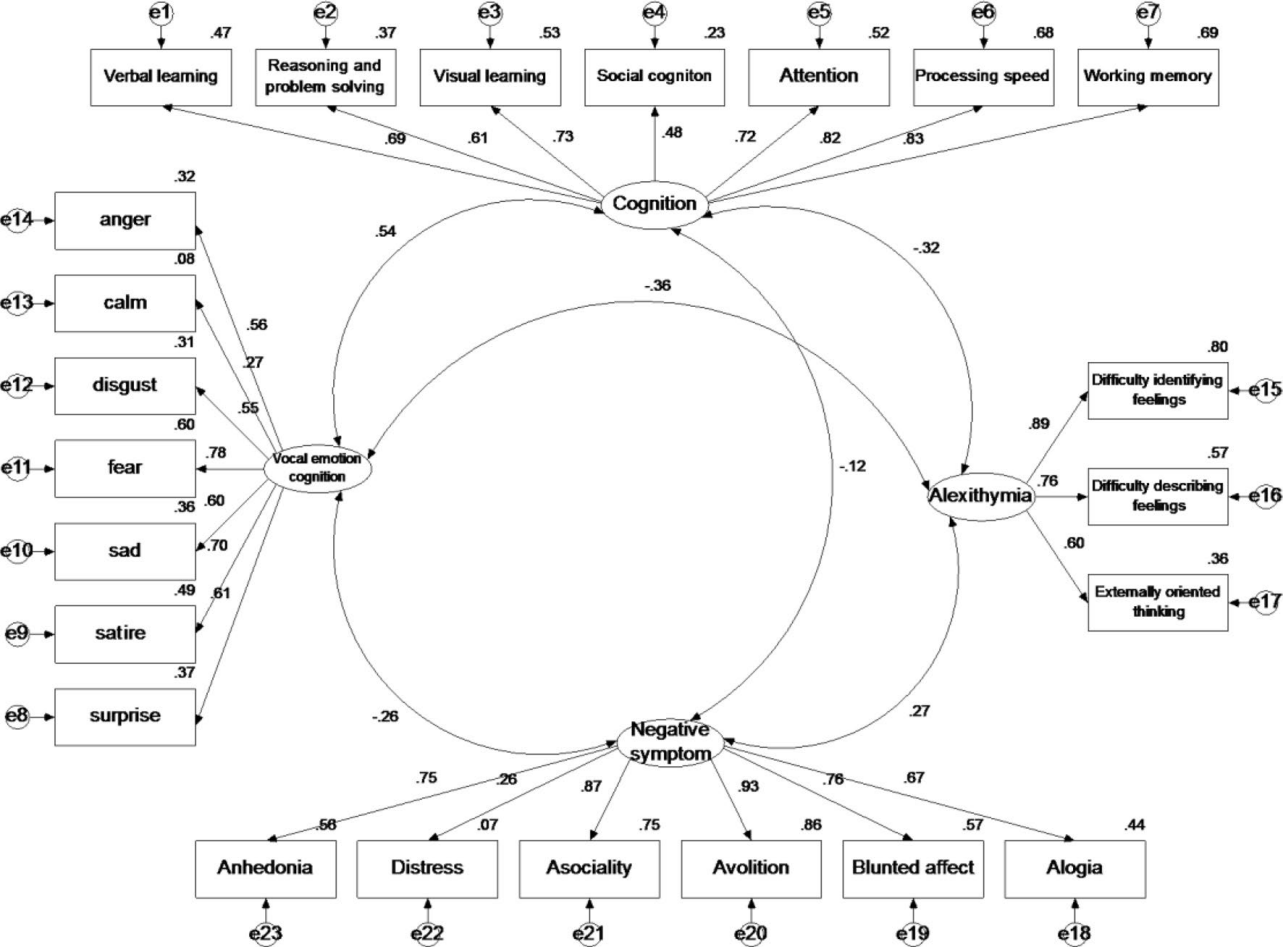
Model 0: Measurement Model. Circles represent unobserved latent variables. Rectangles represent observed measured variables. Values are standardized path coefficients. The figure reflects a measurement model that shows the degree of fit between the four latent variables (neurocognition, vocal emotion cognition, alexithymia, and negative symptoms) and their respective indicators.

Model 0 examined the degree to which the latent variables for neurocognition, vocal emotional cognition, alexithymia, and negative symptoms loaded on their respective indicators (Fig. 1). This first model is essentially a confirmatory factor analysis, and the model fit was extremely good, indicating that the latent variables and indicators were strongly associated.

### Initial model and final model

**Figure 2:**
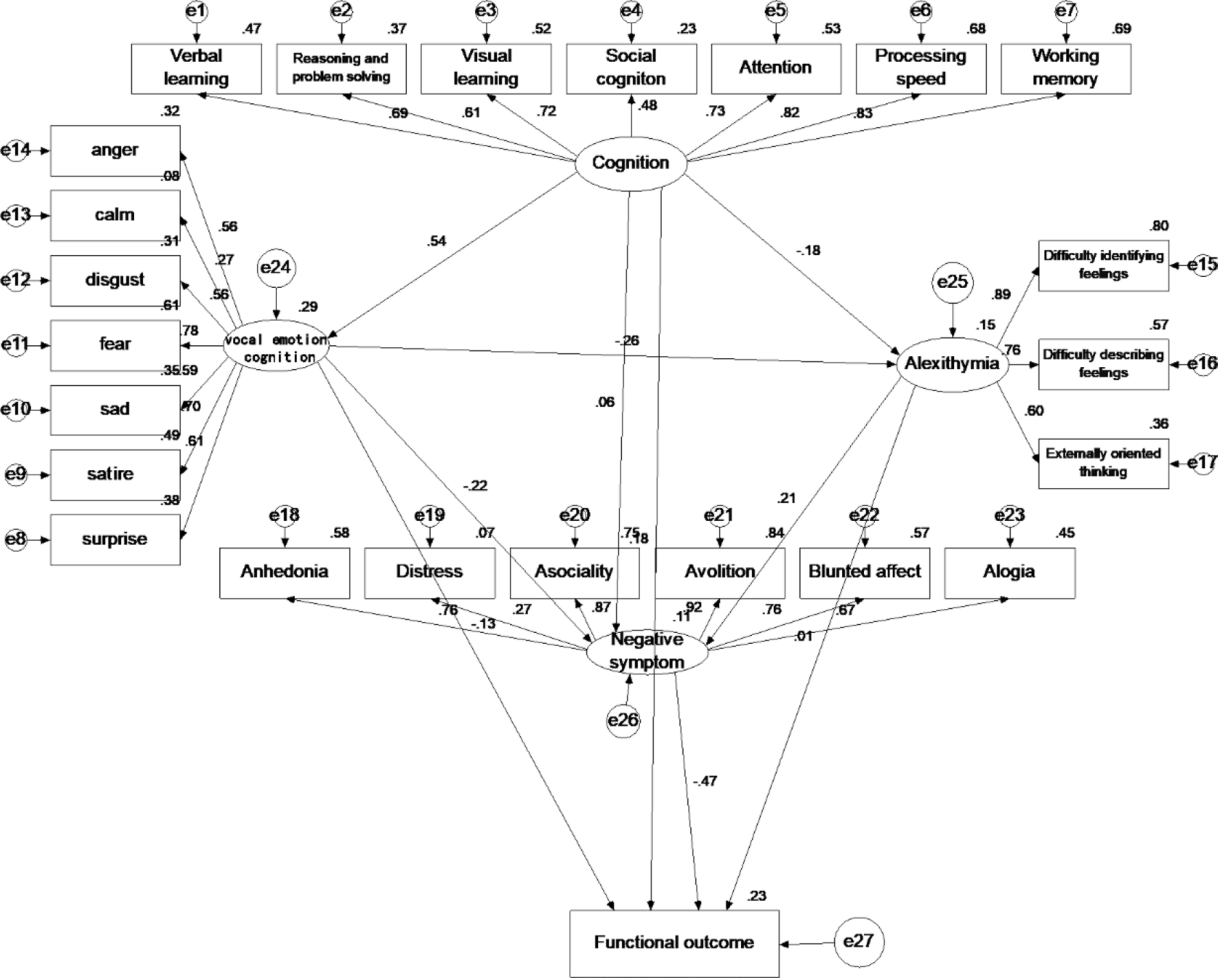
Model 1: Initial Model. This figure is a schematic of the initial, non-trimmed, structural model that includes all of the variables considered.

**Figure 3:**
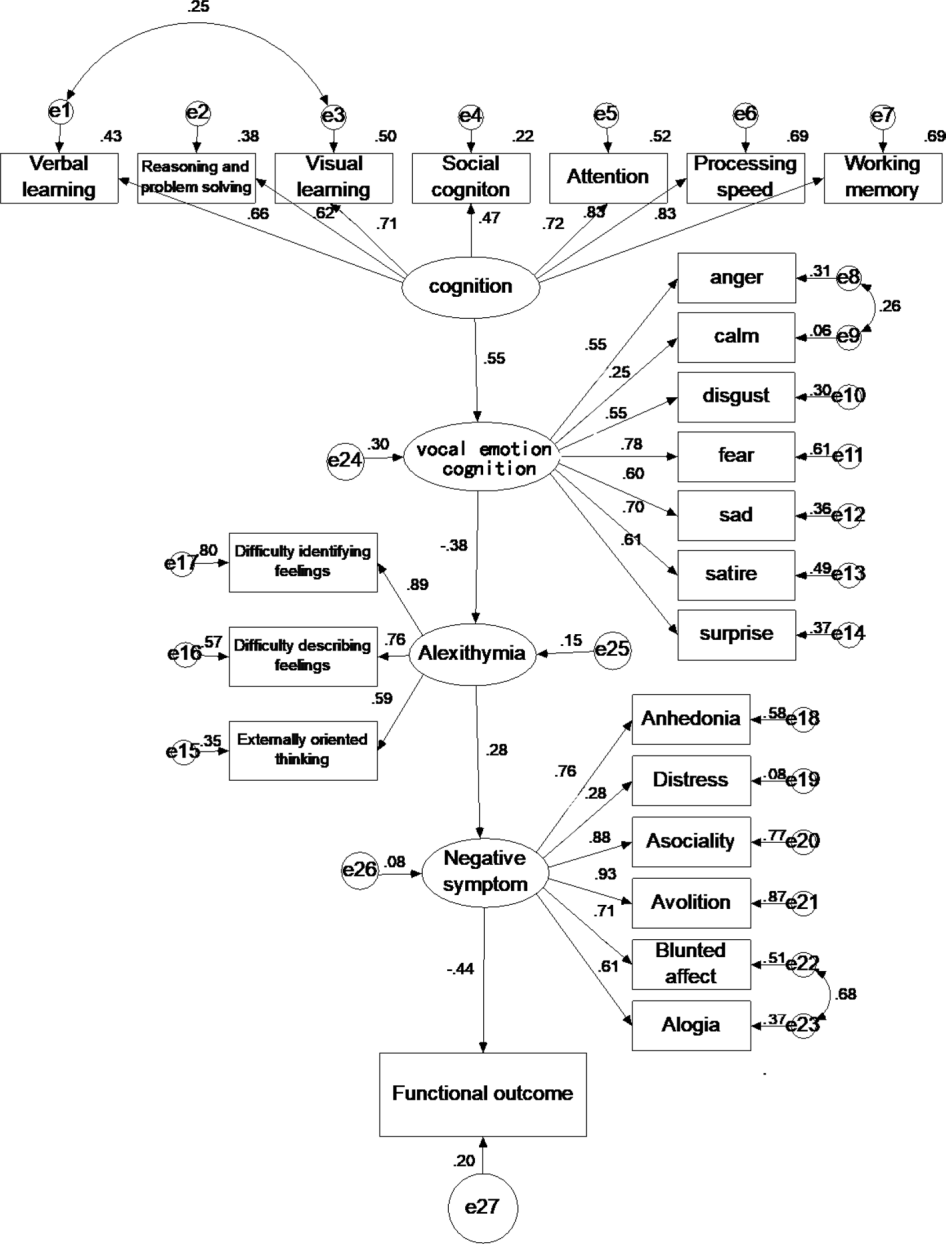
Model 2: Final Model. This figure is the final, trimmed model after modifications. It shows a single path running through cognition, vocal emotion cognition, alexithymia, negative symptom, and functional outcome.

We then added neurocognition, vocal emotion cognition, alexithymia, negative symptom, and functional outcome to create a single path in the model (Fig. 2), and the model fit was good. Next, modification indices from the AMOS software were used to determine some paths that could be discarded to improve the fit. The abovementioned modifications resulted in a more streamlined model that also had a good fit (Fig. 3). The model reflects a relatively linear sequence leading from neurocognition to vocal emotion cognition to alexithymia to negative symptoms and to functional outcome. Compared to the initial model, the final model is more parsimonious (requiring fewer constructs and connections), and the fit indices are slightly higher. Further, given that it is more parsimonious, the model is also more stable. Based on these results, a final trimmed model that uses a single path running from neurocognition to vocal emotion cognition to alexithymia to negative symptoms to functional outcome was selected. This model was chosen based on it being more concise and having better fit indices than the untrimmed model. The statistical results of model fitting degree are shown in Table 2.

**Table 2:** Model fit statistics.

Model	RMSEA	CFI	IFI	TLI	AIC	BIC	Ratio of X^2^
Model 0	0.072	0.884	0.886	0.869	500.096	656.649	1.768
Model 1	0.073	0.873	0.876	0.856	522.380	723.986	1.804
Modified model	0.052	0.937	0.938	0.928	456.880	637.518	1.404
Model 2	0.053	0.934	0.935	0.926	455.437	618.011	1.412

## Discussion

Overall, this study showed that there were deficits in neurocognition, vocal emotional recognition, and alexithymia in the schizophrenia group. Importantly, we evaluated models of outcome in schizophrenia ranging from micro-level neurocognition to macro-level functional outcomes. The observed data fit the proposed bottom-up model. We demonstrated that these variables formed a single pathway, from neurocognition to vocal emotion cognition to alexithymia to negative symptoms to poor functional outcomes.

### Neurocognition

We found that neurocognitive ability, as measured by each dimension of the MCCB, was significantly worse in the schizophrenia group than in the control group. While the underlying causes of these cognitive impairments are unclear, abnormal brain structures and function observed in patients with schizophrenia are believed to play a major role. In fact, observed abnormalities in the white matter^37^ as well as whole brain volume may lead to deficits in abstraction/flexibility, language, and attention/concentration. Furthermore, disproportionally larger lateral ventricles have been associated with poorer psychomotor speed and attention/concentration. Both the prefrontal cortex and the hippocampus have been found to be dysfunctional in schizophrenia patients, and additionally, the prefrontal cortex tends to be associated with measures of executive function and the hippocampus correlates with memory and executive function^38^. Neurocognition is a fundamental cognitive process that can be used as a biomarker for underlying neurodevelopmental problems^8^ that may contribute to higher-level cognitive impairment.

### Vocal emotion cognition

We found that the recognition accuracy for different emotions (anger, disgust, fear, sadness, surprise, and satire, but not for calm) in the schizophrenia group was significantly lower than that in the control group. These results are consistent with previous studies^39^ and are likely because of the interaction between early sensory and late cognitive stage impairments^40^. In fact, some studies have found that schizophrenia patients exhibit a deficit in basic auditory processing^41–43^ and lack cognitive control^40^, resulting in deficits in their ability to recognize information and critical acoustic characteristics based upon tone of voice. Finally, schizophrenia patients tend to show a decreased ability to identify emotional prosody. The amygdala, cerebellum and insula play crucial roles in emotional regulation, perception, and awareness. However, volumes of the left amygdala^44^, bilateral cerebellar cortex^45^, and insula^46^ have been found to be lesser in patients with schizophrenia than in healthy controls. Echoing previous studies, this study also showed that it is most difficult for patients to recognize disgust. It is conceivable that reduced recognition of disgust is a form of self-protection; schizophrenics often face stigma and social exclusion as a result of the disease, and reduced sensitivity to social exclusion signals might protect them from social alienation^47^.

### Alexithymia

The results of this study are consistent with those of previous studies^24,27^ in that the schizophrenia group exhibited greater levels of alexithymia than the control group, characterized by the absence of emotional experience (including the experience’s associated cognitive appraisal). There are cognitive and emotion mechanisms of alexithymia in schizophrenia. For example, difficulty in identifying feelings and increased externally oriented thinking are associated with more neurocognitive deficits, such as slower processing speed^48^, worse attention, and dysfunctional information processing^18^. Furthermore, difficulty in describing feelings can lead to heightened levels of emotional distress^48^.

### The bottom-up causal model of functional outcome in schizophrenia

We demonstrated a single pathway that links neurocognition to functional outcomes; with neurocognition linked to vocal emotion cognition, which is linked to alexithymia, which is linked to negative symptoms, which in turn are linked to functional outcomes.

Previous papers emphasize that ability, beliefs, and motivation mediated the connection between visual processing and functional outcome^28,49^, but did not expand upon the more specific capacity and personality traits, such as emotion cognition, specifically the ability to identify and describe one’s and others’ emotion, which is necessary for schizophrenia patients to integrate into society. Further, they only examined the role of visual processing in the model, ignoring other aspects of basic cognition. Other studies found that poor neurocognitive abilities of schizophrenia patients, such as temporary delay after tone matching^50^, reduced processing speed^51^, poor pre-attentive processes and attention-dependent processes^52,53^, as well as deficits in working memory (interruption of the ability to “maintain”)^54^, lead to impairments in the processing of voice pitch and intensity, which then result in difficulties in emotional recognition. Accordingly, this present study used MCCB to investigate the basic cognitive ability of schizophrenia patients in order to obtain more comprehensive results.

The present study found three mediating paths (one for vocal emotion cognition, one for alexithymia and one for negative symptoms) between the neurocognition and functional outcomes. Specifically, a reduced sensitivity and generally blunted neural response to the emotional qualities and prosody of speech and music make it difficult for individuals to describe and recognize emotions. This is likely the underlying reason for the interpersonal communication problems associated with alexithymia. Furthermore, the low activation of the emotional recognition brain network potentially causes defects in emotional attention and recognition in alexithymia. Emotional identification impairments are a problem of perceptual representation, causing the individual to lose balance with the socio-emotional world, leading to the emergence of pressure and conflict^55^. Alexithymia patients need to involve their cognitive abilities to a greater extent to explain emotional experience, and when the basic emotional processing is defective, this ability to describe emotion is naturally damaged. Subsequently alexithymia results in reduced empathy and an impaired ability to recognize the emotions of others^56^. These reduced abilities lead to frustration in the social environment, which ultimately leads to negative attitudes and self-beliefs. These dysfunctional attitudes in turn contribute to reduced motivation and interest that are clinically considered as negative symptoms^28,57^. Finally, negative symptoms and functional outcome are based on a cascade relationship, in which more serious negative symptoms lead to worse functional outcome in patients with schizophrenia. This is consistent with results from previous reports^32,58^ and was also observed in two longitudinal studies^29,31^. Negative symptoms are an important factor in the overall prognosis of patients with schizophrenia and represent a major hindrance to their return to society^59^. Schizophrenia patients who exhibit more negative symptoms tend to exhibit less interpersonal communication with family members and community members as well as avoidance of family and social functions. They also often show impairment in personal care, self-care, and health. In addition, the chronic process of the disease can also aggravate the impairment of these abilities. Therefore, negative symptoms are direct predictors of functional outcome. Thus, damaged neurocognition causes abnormal vocal emotion recognition, and this abnormal condition leads to damaged emotional expression, which gradually manifest as flat affect in patients, which is the precise core component of negative symptoms.

Importantly, the bottom-up model suggests that it may be possible to intervene in the upstream neurocognitive components in order to prevent the development of social cognition and the negative symptoms, thus interrupting the harmful consequences of this pathway. It may also be possible to develop proper interventions aimed at improving patients’ ability to recognize and articulate their feelings so that schizophrenia patients could ameliorate negative symptoms and restore functional outcomes. The observed complex associations among the investigated factors strongly suggest that multi-domain and multi-level interventions should be provided as a standard treatment to improve the functional outcome^36^.

## Limitation

The current study has some limitations. First, the use of cross-sectional data for analysis weakens causality inference. Additionally, some studies have found that neurocognitive dysfunction^60^, social cognitive impairment^61^, and negative symptoms^31^ are present even in the early stages of schizophrenia, but when exactly each deficit occurs remains unclear. Therefore, future studies using a longitudinal design to determine the temporal relationship between these factors and functional outcomes, and develop specific and effective interventions, are needed. Furthermore, the concept of negative symptoms needs to be further refined. The latent structure of negative symptoms is best conceptualized in relation to the five consensus domains, and this can help make the targeted treatment of schizophrenia more precise and efficient^25,62^. Another limitation is that only 125 patients were assessed, future research should expand sample size to improve the credibility of statistical results.

## Conclusion

The present study demonstrated that cognitive impairment in schizophrenia involves a wide range of non-social and social cognitive fields and clarified the multivariate relationships among neurocognition, vocal emotion cognition, alexithymia, negative symptoms, and functional outcome in schizophrenia. Our results support the hypothesized information processing bottom-up model, whereby neurocognition deficits contribute to vocal emotion cognition impairments and alexithymia, followed by negative symptoms and a reduced functional outcome. Therefore, this integrative model has potential significance in guiding the development of novel treatments and better prognosis for patients with schizophrenia.

## Methods

### Participants

This study included 135 schizophrenia patients (83 males and 52 females) who met the Diagnostic and Statistical Manual of Mental Disorders, Fifth Edition (DSM-V) criteria for schizophrenia and 73 healthy controls (24 males and 49 females) from Beijing HuiLongGuan Hospital. The control participants had no lifetime history of any Axis I disorders or family history of schizophrenia or schizoaffective disorders in their first-degree relatives. The exclusion criteria of all participants included a history of head trauma with loss of consciousness longer than 15 min, substance abuse or dependence within the past 6 months, intellectual disability, debilitating or unstable medical illness, and other neurological diseases. All participants provided written informed consent before undergoing any research procedure. The study protocol was conducted in accordance with the Declaration of Helsinki and was approved by the respective research ethics boards and institutional review boards of Beijing Huilongguan Hospital.

### Neuropsychological and psychopathological assessment

#### MATRICS consensus cognitive battery (MCCB)

Neurocognition was evaluated using the Chinese version of MATRICS (Measurement and Treatment Research to Improve Cognition in Schizophrenia) Consensus Cognitive Battery (MCCB) ^63^, which includes 10 tests measuring 7 domains: speed of processing, attention, working memory, verbal memory, visual memory, reasoning and problem solving, and social cognition. The composite and subtest scores from the MCCB were converted from raw data to T-scores with a mean of 50 and a standard deviation of 10.

#### Vocal emotion cognition test

We selected 2 sets of 56 vocal stimuli pronounced by 4 actors (2 men and 2 women). Seven emotional categories were used in this study (anger, calm, disgust, fear, sadness, surprise, and satire); the recorded contents in mandarin Chinese are as follows: “1. What time is it now; 2. It’s eight o’clock now.” Participants were seated in front of a computer in a quiet room to perform this vocal emotion test, which presented vocal clips in a pseudo-random sequence. All vocal stimuli were played binaurally via stereo headphones. After listening to the voice clip, the subjects were directed to use the mouse to select one of seven progress bars on the screen that represent seven different emotions to judge the emotion type expressed by each voice clip they heard. The value represents the number of correctly recognized vocal emotions.

#### Toronto alexithymia scale (TAS)

We used the 20-item TAS-20 to evaluate 3 subscales of alexithymia: (1) difficulty identifying feelings, (2) difficulty describing feelings, and (3) externally oriented thinking. Each item was rated using a five-point Likert scale (from 1, “strongly disagree,” to 5, “strongly agree”); subscales were computed by summing relevant items and the total alexithymia score by summing responses to all 20 items, with higher scores representing a higher degree of alexithymia. The TAS-20 has demonstrated validity, stability, and reliability^64^.

#### Brief negative symptom scale (BNSS)

The severity of negative symptoms in schizophrenia was evaluated using the BNSS, which had 13 items, including 6 subscales: anhedonia, distress, asociality, avolition, blunted affect, and alogia. Each item was scored using a 7-point scale (0, “normal”; 1, “suspicious”; 2, “mild”; 3, “moderate”; 4, “moderate to severe”; 5, “severe”; 6, “extremely severe”). The score of each dimension is equal to the sum of the items contained, and the total score of the scale is equal to the sum of the points of each dimension. The total score of scale ranged from 0 to 78. The higher the score, the more serious the negative symptoms^65^.

#### Personal and social performance scale (PSP)

The PSP mainly evaluates the function of patients in four aspects, and another total score was assessed according to the scoring criteria. The range of the total score is 0–100 points, which is divided into 10 grades. A total score of 71–100 points indicated that there was no difficulty or only a slight difficulty in social function and interpersonal communication; 31–70 points indicated different degrees of capability defects; and ≤ 30 points indicated that the function was low and the patient needed positive support or close monitoring. In this study, the patient’s functional outcome was evaluated using only the total score ^66^.

#### Data analyses

Data were analysed using IBM SPSS Statistics, Version 23.0 (Armonk, NY, USA), and AMOS 17.0. Eighteen (10 patients and 8 controls) participants were excluded from analyses because of missing data or based on outlier scale scores (> 3 or < 3 standard deviations above the mean). Group differences (patients vs. controls) in demographics, MCCB, vocal emotion cognition task, and TAS scores were examined using an independent sample *t* test for continuous variables and chi-square test for categorical variables. Repeated measure ANOVA for accuracy was conducted with various emotions as the within-subject parameters in patients. Pearson’s correlations were conducted to examine the association between MCCB scores and vocal emotional cognition and TAS and BNSS and PSP.

Structural equation modelling was used to summarize relationships among measures described in the previous sections using latent variables and to test the plausibility of causal associations among these constructs. The analysis included patients only. Our measurement model (M0) consisted of neurocognition, vocal emotion cognition, alexithymia, and negative symptoms. Path analysis was then used to test the relationship between neurocognition, vocal emotion cognition, alexithymia, negative symptoms, and functional outcome. In the analysis, neurocognition was treated as the exogenous variable, whereas vocal emotion cognition, alexithymia, and negative symptoms were treated as the mediating variables.

Model fit was assessed using ranges of acceptable fit values, including comparative fit index (CFI) ≥ 0.90, Tucker-Lewis index (TFI) ≥ 0.90, root mean square error of approximation (RMSEA) ≤ 0.06, and standardized root mean square residual ≤ 0.08, to indicate that the hypothesized model fits the observed data relatively well.

## Data Availability

The datasets generated during and/or analysed during the current study are available from the corresponding author on reasonable request.

## References

[1] Jiménez-López E, Sánchez-Morla EM, López-Villarreal A (2019). Neurocognition and functional outcome in patients with psychotic, non-psychotic bipolar I disorder, and schizophrenia a five-year follow-up. Eur. Psychiatry..

[2] Savla GN, Vella L, Armstrong CC, Penn DL, Twamley EW (2013). Deficits in domains of social cognition in schizophrenia: a meta-analysis of the empirical evidence. Schizophr. Bull..

[3] Wojtalik JA, Smith MJ, Keshavan MS, Eack SM (2017). A systematic and meta-analytic review of neural correlates of functional outcome in schizophrenia. Schizophr. Bull..

[4] Jackson JM (1996). what are the functional consepuences of neurocognitive deficits in schizophrenia?. Am. J. Psychiatry..

[5] Vaskinn A, Sundet K, Friis S (2008). Emotion perception and learning potential: Mediators between neurocognition and social problem-solving in schizophrenia?. J. Int. Neuropsychol. Soc..

[6] Brekke J, Kay DD, Lee KS, Green MF (2005). Biosocial pathways to functional outcome in schizophrenia. Schizophr. Res..

[7] Schmidt SJ, Mueller DR, Roder V (2011). Social cognition as a mediator variable between neurocognition and functional outcome in schizophrenia: Empirical review and new results by structural equation modeling. Schizophr. Bull..

[8] Melle I (2019). Cognition in schizophrenia: a marker of underlying neurodevelopmental problems?. World Psychiatry.

[9] Bell M, Tsang HWH, Greig TC, Bryson GJ (2009). Neurocognition, social cognition, perceived social discomfort, and vocational outcomes in schizophrenia. Schizophr Bull..

[10] Mark J, Rassovsky Y, Keith H, Michael F (2006). Social Perception as a Mediator of the Influence of Early Visual Processing on Functional Status in Schizophrenia. Am. J. Psychiatry..

[11] Oliver LD, Haltigan JD, Gold JM (2018). Lower- and higher-level social cognitive factors across individuals with schizophrenia spectrum disorders and healthy controls: relationship with neurocognition and functional outcome. Schizophr Bull..

[12] Fett AKJ, Viechtbauer W, de Dominguez MG, Penn DL, van Os J, Krabbendam L (2011). The relationship between neurocognition and social cognition with functional outcomes in schizophrenia: a meta-analysis. Neurosci Biobehav Rev..

[13] Green MF, Horan WP, Lee J (2019). Nonsocial and social cognition in schizophrenia: current evidence and future directions. World Psychiatry.

[14] Schirmer A (2018). Is the voice an auditory face? An ALE meta-analysis comparing vocal and facial emotion processing. Soc. Cogn. Affect Neurosci..

[15] Morningstar M, Nelson EE, Dirks MA (2018). Maturation of vocal emotion recognition: Insights from the developmental and neuroimaging literature. Neurosci. Biobehav. Rev..

[16] Mitchell RLC, Ross ED (2013). Attitudinal prosody: What we know and directions for future study. Neurosci. Biobehav. Rev..

[17] Cheang HS, Pell MD (2008). The sound of sarcasm. Speech Commun..

[18] Gawęda Ł, Krężołek M (2019). Cognitive mechanisms of alexithymia in schizophrenia: Investigating the role of basic neurocognitive functioning and cognitive biases. Psychiatry Res..

[19] Yu S, Li H, Liu W (2011). Alexithymia and personality disorder functioning styles in paranoid schizophrenia. Psychopathology.

[20] Parker PD, Prkachin KM, Prkachin GC (2005). Processing of facial expressions of negative emotion in alexithymia: The influence of temporal constraint. J. Pers..

[21] Goerlich KS, Witteman J, Aleman A, Martens S (2011). Hearing feelings: Affective categorization of music and speech in Alexithymia, an ERP study. PLoS ONE.

[22] Goerlich-Dobre KS, Witteman J, Schiller NO, van Heuven VJP, Aleman A, Martens S (2014). Blunted feelings: Alexithymia is associated with a diminished neural response to speech prosody. Soc. Cogn. Affect Neurosci..

[23] Swart M, Kortekaas R, Aleman A (2009). Dealing with feelings: characterization of trait Alexithymia on emotion regulation strategies and cognitive-emotional processing. PLoS ONE.

[24] Ospina LH, Shanahan M, Perez-Rodriguez MM, Chan CC, Clari R, Burdick KE (2019). Alexithymia predicts poorer social and everyday functioning in schizophrenia and bipolar disorder. Psychiatry Res..

[25] Strauss GP, Nuñez A, Ahmed AO (2018). The latent structure of negative symptoms in schizophrenia. JAMA Psychiat..

[26] Balogh N, Égerházi A, Berecz R, Csukly G (2014). Investigating the state-like and trait-like characters of social cognition in schizophrenia: a short term follow-up study. Schizophr. Res..

[27] Tang XW, Yu M, Duan WW (2016). Facial emotion recognition and alexithymia in Chinese male patients with deficit schizophrenia. Psychiatry. Res..

[28] Green MF, Hellemann G, Horan WP, Lee J, Wynn JK (2012). From perception to functional outcome in schizophrenia. Arch. Gen. Psychiatry..

[29] Fervaha G, Foussias G, Agid O, Remington G (2014). Motivational and neurocognitive deficits are central to the prediction of longitudinal functional outcome in schizophrenia. Acta Psychiatr. Scand..

[30] Yung AR, Cotter J, Wood SJ (2015). Childhood maltreatment and transition to psychotic disorder independently predict long-term functioning in young people at ultra-high risk for psychosis. Psychol. Med..

[31] Yung AR, Nelson B, McGorry PD, Wood SJ, Lin A (2018). Persistent negative symptoms in individuals at Ultra High Risk for psychosis. Schizophr. Res..

[32] Villalta-Gil V, Vilaplana M, Ochoa S (2006). Neurocognitive performance and negative symptoms: are they equal in explaining disability in schizophrenia outpatients?. Schizophr. Res..

[33] Strauss GP, Sandt AR, Catalano LT, Allen DN (2012). Negative symptoms and depression predict lower psychological well-being in individuals with schizophrenia. Compr. Psychiatry..

[34] Strauss GP, Harrow M, Grossman LS, Rosen C (2010). Periods of recovery in deficit syndrome schizophrenia: a 20-year multi-follow-up longitudinal study. Schizophr. Bull..

[35] Nelson BD, Bjorkquist OA, Olsen EK, Herbener ES (2015). Schizophrenia symptom and functional correlates of anterior cingulate cortex activation to emotion stimuli: An fMRI investigation. Psychiatry Res. Neuroimaging..

[36] Galderisi S, Rossi A, Rocca P (2014). The influence of illness-related variables, personal resources and context-related factors on real-life functioning of people with schizophrenia. World Psychiatry.

[37] Zhang X, Yang M, Du X (2019). Glucose disturbances, cognitive deficits and white matter abnormalities in first-episode drug-naive schizophrenia. Mol Psychiatry..

[38] Antonova E, Sharma T, Morris R, Kumari V (2004). The relationship between brain structure and neurocognition in schizophrenia: a selective review. Schizophr. Res..

[39] Lin Y, Ding H, Zhang Y (2018). Emotional prosody processing in schizophrenic patients: a selective review and meta-analysis. J. Clin. Med..

[40] Dondaine T, Robert G, Péron J (2014). Biases in facial and vocal emotion recognition in chronic schizophrenia. Front Psychol..

[41] Kantrowitz JT, Leitman DI, Lehrfeld JM (2013). Reduction in tonal discriminations predicts receptive emotion processing deficits in schizophrenia and schizoaffective disorder. Schizophr. Bull..

[42] Thomas ML, Green MF, Hellemann G (2017). Modeling deficits from early auditory information processing to psychosocial functioning in schizophrenia. JAMA Psychiat..

[43] Pinheiro AP, Del Re E, Mezin J (2013). Sensory-based and higher-order operations contribute to abnormal emotional prosody processing in schizophrenia: An electrophysiological investigation. Psychol. Med..

[44] Mahon PB, Lee DS, Trinh H (2015). Morphometry of the amygdala in schizophrenia and psychotic bipolar disorder. Schizophr. Res..

[45] Laidi C, d’Albis MA, Wessa M (2015). Cerebellar volume in schizophrenia and bipolar I disorder with and without psychotic features. Acta Psychiatr Scand..

[46] Shepherd AM, Matheson SL, Laurens KR, Carr VJ, Green MJ (2012). Systematic meta-analysis of insula volume in schizophrenia. Biol. Psychiatry..

[47] Lindner C, Dannlowski U, Walhöfer K (2014). Social alienation in schizophrenia patients: Association with insula responsiveness to facial expressions of disgust. PLoS ONE.

[48] Fogley R, Warman D, Lysaker PH (2014). Alexithymia in schizophrenia: Associations with neurocognition and emotional distress. Psychiatry Res..

[49] Rassovsky Y, Horan WP, Lee J, Sergi MJ, Green MF (2011). Pathways between early visual processing and functional outcome in schizophrenia. Psychol. Med..

[50] Javitt DC (2009). When doors of perception close: bottom-up models of disrupted cognition in schizophrenia. Annu. Rev. Clin. Psychol..

[51] Mangelinckx C, Belge JB, Maurage P, Constant E (2017). Impaired facial and vocal emotion decoding in schizophrenia is underpinned by basic perceptivo- motor deficits. Cogn. Neuropsychiatry..

[52] Sossong A (2013). Relationship between auditory processing and affective prosody in schizophrenia. Schizophr. Res..

[53] Kantrowitz JT, Hoptman MJ, Leitman DI (2015). Neural substrates of auditory emotion recognition deficits in schizophrenia. J. Neurosci..

[54] Anticevic A, Corlett PR (2012). Cognition-emotion dysinteraction in schizophrenia. Front Psychol..

[55] Prkachin GC, Casey C, Prkachin KM (2009). Alexithymia and perception of facial expressions of emotion. Pers. Individ. Dif..

[56] Bird G, Cook R (2013). Mixed emotions: the contribution of alexithymia to the emotional symptoms of autism. Transl. Psychiatry..

[57] Rector NA, Beck AT, Stolar N (2005). The negative symptoms of schizophrenia: a cognitive perspective. Can. J. Psychiatry..

[58] Foti D, Perlman G, Bromet EJ (2020). Pathways from performance monitoring to negative symptoms and functional outcomes in psychotic disorders. Psychol. Med..

[59] Deserno L, Heinz A, Schlagenhauf F (2016). Computational approaches to schizophrenia: a perspective on negative symptoms. Schizophr. Res..

[60] Bora E, Lin A, Wood SJ, Yung AR, Mcgorry PD, Pantelis C (2014). Cognitive deficits in youth with familial and clinical high risk to psychosis: a systematic review and meta-analysis. Acta Psychiatr. Scand..

[61] Lee TY, Bin HS, Shin NY, Kwon JS (2015). Social cognitive functioning in prodromal psychosis: meta-analysis. Schizophr Res..

[62] Strauss GP, Ahmed AO, Young JW, Kirkpatrick B (2019). Reconsidering the latent structure of negative symptoms in schizophrenia: a review of evidence supporting the 5 consensus domains. Schizophr. Bull..

[63] Yizhuang Z, Jiefeng C, Jiang W (2009). clinical reliability and validity of the chinese version of measurement and treatment research to improve cognition in schizophrenia consensus cognitive battery. Chin. J. Psychiatry..

[64] Bagby RM, Taylor GJ, Parkers JDA (1994). THE TWENTY-ITEM CONVERGENT, TORONTO VALIDITY DISCRIMINANT, AND CONCURRENT twenty-item Toronto Alexithymia Scale ( TAS-20) is a self-report measure of the alexithymia construct that was developed in an attempt to improve the psychometric properties of t. J. Psychosom Res..

[65] Kirkpatrick B, Strauss GP, Nguyen L (2011). The brief negative symptom scale: psychometric properties. Schizophr. Bull..

[66] Nasrallah H, Morosini PL, Gagnon DD (2008). Reliability, validity and ability to detect change of the personal and social performance scale in patients with stable schizophrenia. Psychiatr. Res..

